# Does Human Body Odor Represent a Significant and Rewarding Social Signal to Individuals High in Social Openness?

**DOI:** 10.1371/journal.pone.0094314

**Published:** 2014-04-09

**Authors:** Katrin T. Lübke, Ilona Croy, Matthias Hoenen, Johannes Gerber, Bettina M. Pause, Thomas Hummel

**Affiliations:** 1 Department of Experimental Psychology, University of Düsseldorf, Düsseldorf, Germany; 2 Department of Otorhinolaryngology, University of Dresden Medical School, Dresden, Germany; 3 Department of Neuroradiology, University of Dresden Medical School, Dresden, Germany; West China Hospital of Sichuan University, China

## Abstract

Across a wide variety of domains, experts differ from novices in their response to stimuli linked to their respective field of expertise. It is currently unknown whether similar patterns can be observed with regard to social expertise. The current study therefore focuses on social openness, a central social skill necessary to initiate social contact. Human body odors were used as social cues, as they inherently signal the presence of another human being. Using functional MRI, hemodynamic brain responses to body odors of women reporting a high (n = 14) or a low (n = 12) level of social openness were compared. Greater activation within the inferior frontal gyrus and the caudate nucleus was observed in high socially open individuals compared to individuals low in social openness. With the inferior frontal gyrus being a crucial part of the human mirror neuron system, and the caudate nucleus being implicated in social reward, it is discussed whether human body odor might constitute more of a significant and rewarding social signal to individuals high in social openness compared to individuals low in social openness process.

## Introduction

Across a wide variety of domains, experts differ from novices in their response to stimuli linked to their respective field of expertise. These differences, apparent in overt behavior, are correlated with differential central nervous processing patterns in experts versus novices. For example, when presented with expertise linked stimuli, athletes show stronger activation within task related brain areas compared to novices [1]–[3]. Similar results have been reported when comparing chess masters to chess novices [4], or professional musicians to musical lay persons [5]. Similar differences between “experts” and “novices” can be expected within the domain of social skills. However, whenever social expertise is reported to affect responses to social stimuli, “normal” control groups are compared to individuals featuring social deficits, such as patients suffering from schizophrenia, or autism spectrum disorders [6]. How social expertise affects brain activation in response to social stimuli when otherwise normal individuals with social skills below average are compared to social experts is currently unknown.

Social expertise, or social competence, can be defined as being able to correctly identify and interpret social and emotional information, being highly sensitive to socio-emotional information, being able to memorize social information, and being able to manage social and emotional situations (for an overview see [7]). Importantly, in order to establish social contacts, being socially open is a central skill for socially competent people. Following Kanning's model of social skills, “social openness” (German “Offensivität”, [8]) is characterized by being outgoing and sociable, but also being assertive, decisive, and able to negotiate social conflicts without intentionally causing them. Individuals who describe themselves as high in social openness display a pervasive drive and the necessary skills to initiate and maintain social contact. So far, imaging studies have linked both empathy and social reward sensitivity to brain areas subserving the perception and integration of social information [9]–[11], as well as the processing of social reward [10], [11]. Kaplan and Jacoboni [9] interpreted their findings as suggesting a close link between social competence and mirror neuron system activity. Moreover, similar to social openness, social reward sensitivity, as examined in [10] and [11], reflects the individual disposition to social relationships.

Human body odor represents a ubiquitous and ancient social signal, linked to the domain of social expertise. Humans permanently produce and perceive body odor, and its social and emotional content cannot be manipulated (for reviews on human chemosensory communication see [12]–[15]). It inherently signals the presence of another individual, and has been shown to carry a diversity of social information, ranging from individual identity [16], [17] to transiently experienced affect [18]–[20]. Social expertise seemingly affects responses to chemosensory social stimuli, as social anxiety modulates the central nervous processing of human body odors [21], [22] as well as motor behavior in the context of human body odors [23]. Social anxiety itself is tightly linked to deficits in social skills [24], causing deficits in social interaction performance [25], [26]. Emotionally highly competent individuals, on the other hand, presumably also tending to be socially skilled, outperform less emotionally adept individuals in identifying familiar persons by their body odor [27].

The current study was designed to examine effects of social expertise, precisely social openness, on hemodynamic brain responses to social stimuli, using human body odor as the most basic social stimulus. Comparable to studies in other fields of expertise, an experimental approach comparing highly socially open individuals (“social experts”) with individuals low in social openness (“social novices”) was chosen. Effects of social openness are expected to be most prominent within brain areas involved in social information and reward processing: When presented with human body odor, highly socially open individuals should show stronger hemodynamic responses in these brain regions than individuals low in social openness.

## Materials and Methods

### Ethics statement

Participants gave written informed consent and were paid for their participation. The current study, including the sweat sampling procedure, was carried out in accordance with the Declaration of Helsinki and was approved by the University of Dresden Medical Faculty Ethics Review Board.

### Participants

Twenty-six right-handed women (mean age: 23.0 years, SD = 2.2, range 18–27) of European descent participated in the current study. Only women were recruited due to their overall greater olfactory abilities compared to men [28], and especially due to their higher sensitivity regarding chemosensory social cues [21], [29], [30]. None of these women reported a history of chronic medication, of neurological, psychiatric, major endocrine or immunological diseases or diseases related to the upper respiratory tract. All participants showed normal olfactory abilities (as tested with the “Sniffin' Sticks” test kit, [31], [32]).

In order to identify individuals who would qualify as having a high level of social expertise, and individuals displaying a low level of social expertise, the subscale “Openness” of the “Inventar Sozialer Kompetenzen” (ISK, [8]), a German inventory for the assessment of social skills was used. Within the ISK short version, which was used within the current study, the subscale “Openness” consists of 8 items, such as “It is quite easy for me to quickly get in with a new group of people.” (German: “Es fällt mir sehr leicht, in einer neuen Gruppe schnell Anschluss zu finden.“), or „I always approach people if I want to get to know them.” (German “Ich gehe immer auf Menschen zu, wenn ich sie kennen lernen möchte.“). Each item is phrased as a statement, and participants are asked to indicate their level of agreement on a scale ranging from 1 ( =  “totally disagree”) to 4 ( =  “totally agree”). Conceptually, social openness is related to extraversion, as well as self-confidence and assertiveness, and socially open individuals have been shown to be attentive towards the behavior of others in social interactions [8].

In order to recruit participants representing the two experimental groups of “High Level of Openness” (HO; n = 14) and “Low Level of Openness” (LO; n = 12), applicants answered the ISK during individual preparatory meetings. Those participants scoring higher than mean standard score (M = 100) plus one standard deviation (SD = 10; standard score >110) on “Openness” were identified to belong to HO, whereas participants scoring lower than mean minus one standard deviation (standard score <90) were identified to belong to LO. Applicants whose “Openness” scores did not meet these criteria (n = 43) were excluded from participation and thus not invited to the separately scheduled scanning session (see Table 1 for a distribution of “Openness” scores across included and excluded participants). The resulting extreme groups included participants either belonging to the 15.8% highest ranking or to the 15.8% lowest ranking individuals in “Openness” within the population. This approach ensured a high level of statistical power by spanning a wide range of the independent variable, which is especially important in studies with an exploratory character. According to this selection procedure, HO participants (M = 113.86, SD = 3.09) displayed higher “Openness” scores than LO participants [M = 86.33, SD = 3.73; t(24) = 20.62, p<0.001]. HO and LO participants did not differ in age [t(24) = 0.450, p = 0.656].

**Table 1:** Distribution of “Openness” standard scores across included and excluded participants.

Participants	„Openness“ standard score	n	M ± SD “Openness” standard score
Excluded Participants	92	3	101.47±5.32
	95	5	
	96	2	
	97	2	
	98	3	
	101	9	
	103	4	
	106	8	
	109	7	
„Low Level of Openness“	78	1	86.33±3.73
	83	3	
	86	1	
	89	7	
„High Level of Openness“	112	10	113.86±3.09
	118	3	
	120	1	

### Chemosensory stimuli

Axillary sweat was sampled from 8 male and 8 female students. These donors were on average 22.5 years old (SD = 2.5, range  = 20–30). Male and female donors did not differ in age [t(14) = 0.39, p = 0.704]. All donors reported being of European origin, and denied any acute or chronic medication. Furthermore, no donor indicated suffering from any neurological, psychiatric, endocrine, or immunological disease, or using drugs. Their body-mass-index ranged from 19.3 to 26.0 kg/m^2^ (M = 22.5, SD = 1.9), and all of them were non-smokers. Female donors reported having a regular menstrual cycle and denied use of hormonal contraception.

The donors were instructed to refrain from eating garlic, onions, asparagus, or any other spicy or aromatic food during the 24 hours prior to the odor donation. They were further advised to refrain from using deodorants within this timeframe, and to wash their armpits exclusively with an odorless medical soap (Eubos, Dr. Holbein GmbH, Germany). Male as well as female donors shaved their armpits one day prior to the odor donation. For collecting the axillary odors, one cotton pad was fixed in each of the donor's armpits. The axillary odors were sampled during sleep over the course of one night (sampling duration: M = 8.5 h, SD = 1.0 h). All donors gave written informed consent and were paid for their donation. None of the odor donors acted as a participant within the current study.

Following the completion of collection, cotton pads carrying the sweat samples were chopped and pooled with respect to the donor's sex, then divided into small portions of 0.6 g cotton pad and stored at −20°C. Additionally, samples of pure, unused cotton pads were treated the exact same way to provide for baseline measurements within the fMRI sessions.

### Stimulus Presentation

**Figure 1:**
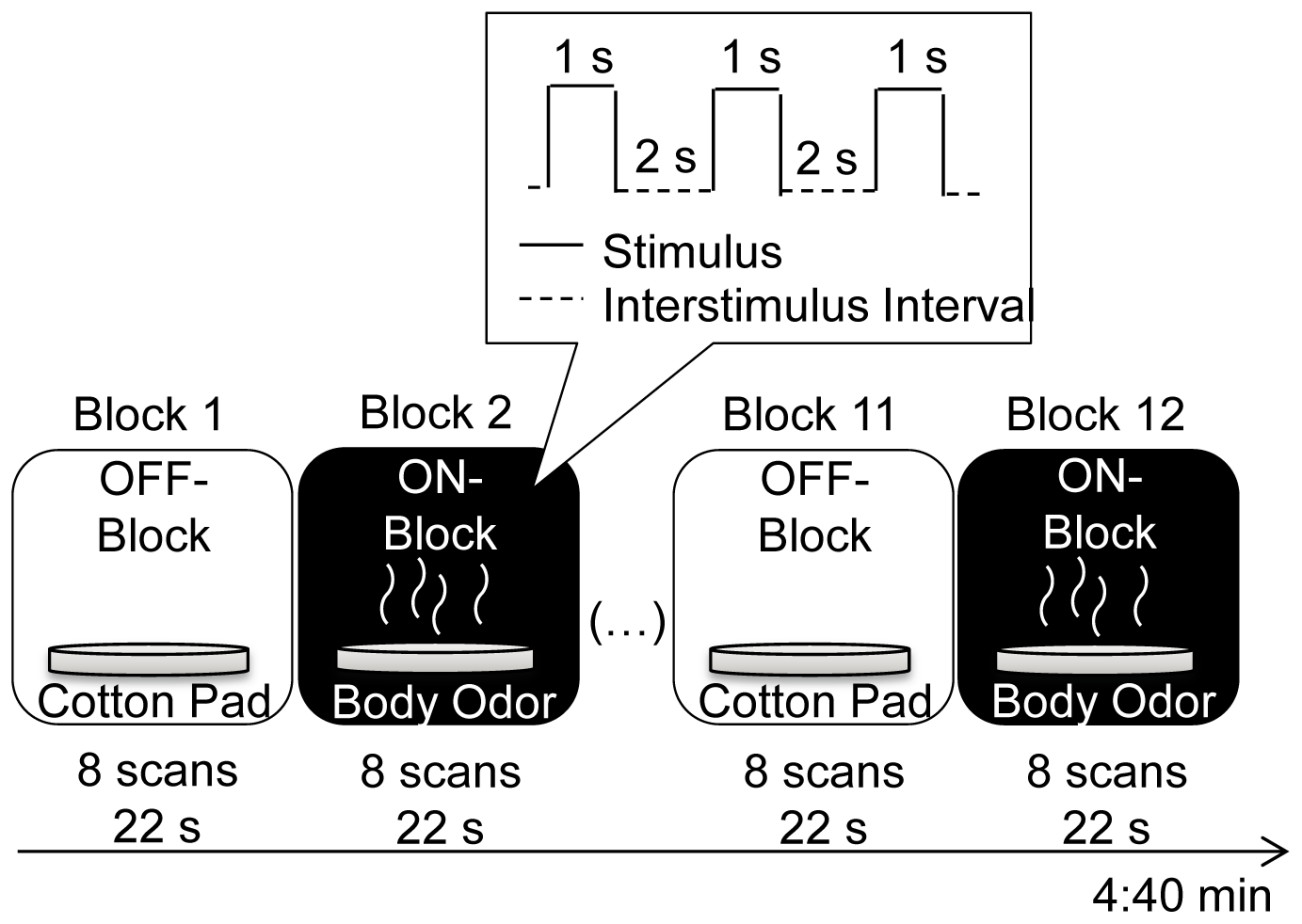
Schematic time course of a scanning session. The participants underwent 4 scanning sessions in succession. Each session consisted of 6 ON-blocks (with presentation of body odor) and 6 OFF-blocks (with presentation of odor-free, pure cotton pad), resulting in a total of 12 blocks. During each block, the stimuli were presented for a period of 1 s with an interstimulus interval of 2 s. Each block had a duration of 22 s, during which 8 scans were conducted.

Participants underwent four scanning sessions in total. In two of these sessions they were presented with male body odors, while in the other two they were presented with female body odors. The order of the scanning sessions was counterbalanced across groups. A self-constructed olfactometer delivered odor pulses embedded in a constant flow of humidified, odorless air in order to avoid any mechanical stimulation. The odors were presented birhinally and intranasally (inner diameter of the Teflon tubing: 4 mm), with a total airflow of 2 liter per minute. Further, the odors were delivered non-synchronously to breathing, as participants performed the velopharyngeal closure technique [33]. The body odors (during ON blocks) as well as the odor-free, pure cotton pads (during OFF blocks) were presented for a period of 1 second with an interstimulus interval of 2 seconds (see Fig. 1).

Participants were not cued for stimulus presentation, and were not asked to perform any detection or other cognitive tasks. Following each session, however, participants were asked to rate the odor's intensity (0 =  not perceivable; 10 =  extremely intense) and hedonic quality (−5 =  extremely unpleasant; 5 =  extremely pleasant).

### fMRI Protocol

A 1.5 T scanner (SONATA-MR, Siemens, Erlangen, Germany) was used for fMRI data acquisition. For functional data 96 volumes per session were acquired by means of a 33 axial-slice matrix 2D SE/EP sequence. Scan parameters included a 192×192 mm^2^ field of view, a TR of 2500 ms, a TE of 40 ms, a 64×64 matrix, a 90° flip angle, a slice thickness of 3 mm, and a voxel size of 3×3×3.75 mm^3^. Additionally, T1-weighted images were acquired using a 3D IR/GR sequence (TR: 2180 ms/TE: 3.39 ms) to localize activated areas. Eight dummy scans were conducted at the beginning of each session to allow the magnetization to reach magnetic equilibrium. Utilizing a block design, in each session the participants received 8 scans during the 22 s ON blocks and 8 scans during the 22 s OFF blocks (see Fig. 1). ON and OFF blocks were repeated 6 times in alternation. Each session lasted 4∶40 minutes.

### fMRI Data Analysis

Preprocessing and statistical analysis were performed using the statistical parametric mapping software package (SPM8, Wellcome Trust Centre for Neuroimaging, London; www.fil.ion.ucl.ac.uk/spm) implemented in Matlab R2010b (Math Works Inc., Natick, MA; USA). Head motions across time were corrected by realigning all scans to the first volume. Participants' T1-weighted images were co-registered to the corresponding mean EPI images and subsequently normalized to Montreal Neurological Institute (MNI) standard space using the segmentation procedure. EPI images were then normalized using the parameters written during segmentation of co-registered T1-weighted images and spatially smoothed using an isotropic Gaussian kernel at 6 mm full width at half maximum.

The responses to male and female body odors were combined for analyses, as the current literature does not provide data that would allow for specific sex-related hypotheses. Both body odors were presented during scanning in order to prevent any bias that might result from processing of same-sex vs. other-sex body odors. However, the odors were not combined during scanning to avoid creating an artificial chemosignal.

In order to identify the effects of the body odor presentation, first level linear contrast images were entered into a general linear model, applying a canonical hemodynamic response function. Statistical parametrical maps were generated for each participant. The parameters written during realignment were entered as multiple regressors to capture residual movement artifacts. A high-pass filter of 128 ms was applied in order to exclude variance due to aliasing. In order to examine the effects of the level of social openness, the resulting contrast images were analyzed using a two-sample-t-test.

Further, in order to test whether the presentation of body odors in general activated brain areas reported to be involved in the processing of complex social chemosignals (for reviews see [12], [14]), a second level analysis across all participants, using a one sample t-test, was performed. These networks include the fusiform cortex [19], [34], the anterior and posterior cingulate cortex [19], [35], [36], and the insular cortex [19], [36]. Accordingly, a Region of Interest Analysis was performed for those regions. Masks were created using the WFU Pick Atlas 3.0.3 [37], [38] toolbox for SPM. The statistical threshold was set at p<0.001 (uncorrected), and the minimum cluster size was set at k = 20. The coordinates of the activation are presented according to MNI.

## Results

**Figure 2:**
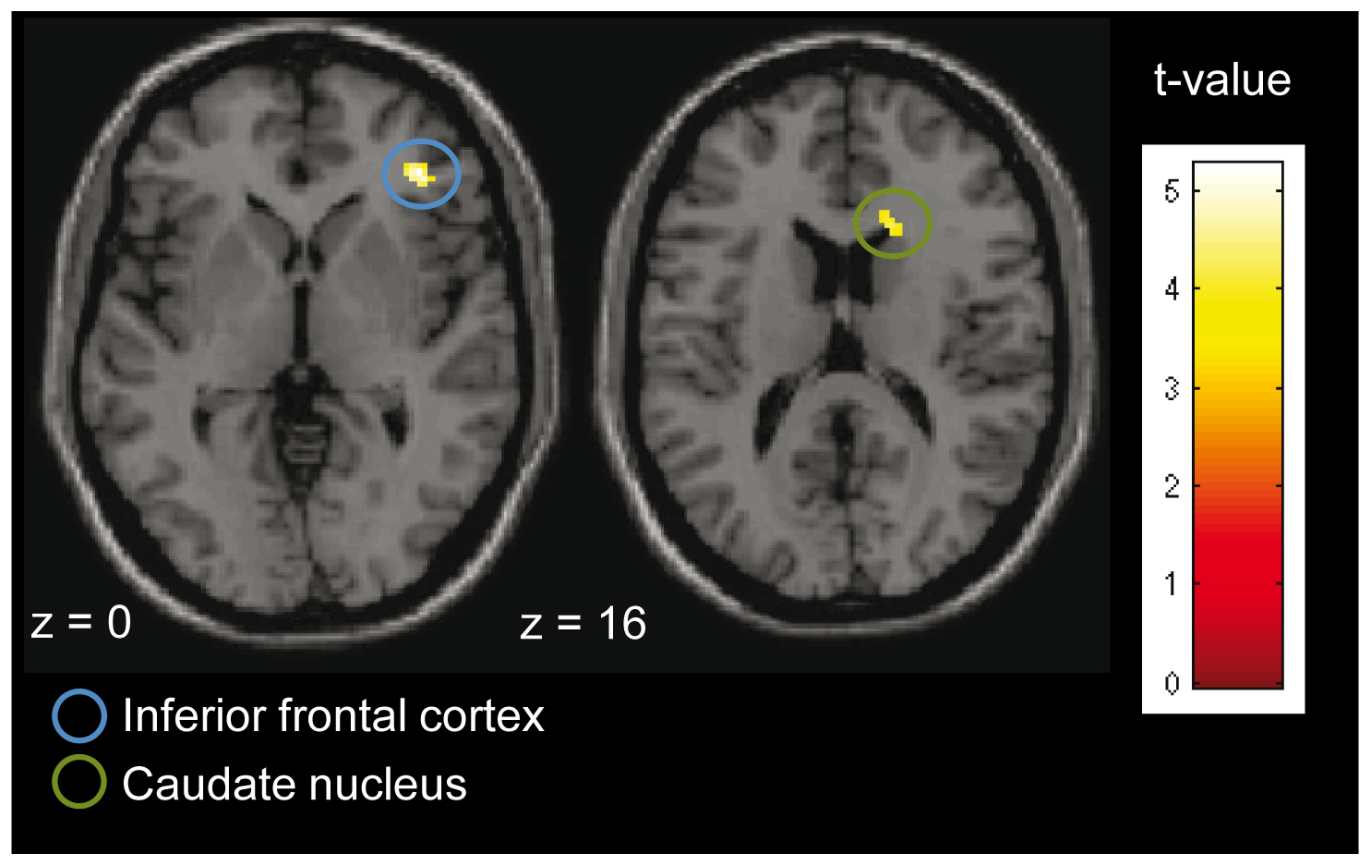
Activation in response to body odors in HO vs. LO participants. HO participants show activation within the right inferior frontal cortex and within the right caudate nucleus (k≥20; p<0.001). For visualization a normalized template provided by SPM 8 software (single_subj_T1.nii) was used.

**Figure 3:**
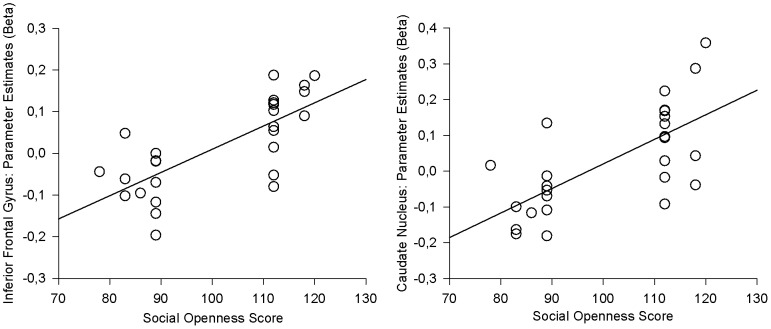
Individual beta values of peak activation plotted against social openness scores. Left paragraph: Peak activation within the inferior frontal gyrus vs. social openness scores; right paragraph: Peak activation within the caudate nucleus vs. social openness scores.

The hemodynamic brain response to the body odors presented indeed varied with social openness. Comparing the parameter estimates of the first level ON-OFF-contrasts in HO versus LO participants showed greater activation within the right inferior frontal gyrus (peak located at x = 40/y = 38/z = 0; t = 5.26; cluster size 37, see Fig. 2), and within the right caudate nucleus (peak located at x = 16/y = 22/z = 14; t = 4.28; cluster size 33, see Fig. 2) in HO compared to LO participants. The reverse (LO vs. HO) contrast did not yield any suprathreshold activation. Further, significant linear relationships between individual beta values and social openness scores were observed: Social openness scores were highly positively correlated with both peak activation within the right inferior frontal gyrus (r = 0.715, p<0.001, see Fig. 3, left paragraph) and the right caudate nucleus (r = 0.685, p<0.001, see Fig. 3, right paragraph).

**Figure 4:**
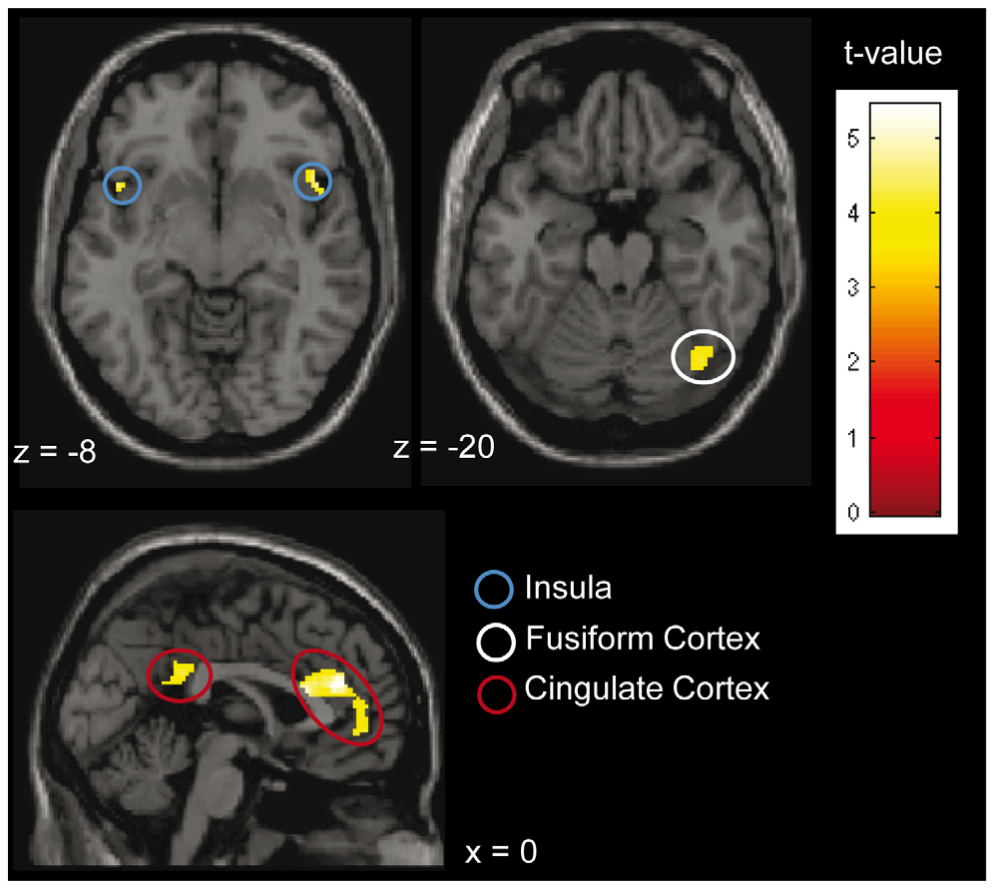
Activation in response to the body odors across all participants (n = 26). Parameters: k≥20; p<0.001; contrast: ON vs. OFF. For visualization a normalized template provided by SPM 8 software (single_subj_T1.nii) was used.

Contrasting the perception of body odors (ON) with the perception of pure cotton pad (OFF) across all participants, using the specified masks, significant activation within the fusiform cortex, the anterior and posterior cingulate cortex and the insular cortex was evident (see Table 2, Fig. 4). The reverse contrast did not yield any suprathreshold activation.

**Table 2:** Significant peaks for body odor perception across all participants (n = 26) in areas reported to be involved in body odor processing.

			MNI coordinates		
	Cluster size	t-value	x	y	z
Fusiform Cortex	21	4.36	−30	−10	−30
	35	4.12	40	−68	−20
Anterior Cingulate Cortex	461	5.43*	0	38	22
		5.14*	−2	22	20
		4.08*	0	50	−2
Posterior Cingulate Cortex	155	5.38*	8	−38	8
		4.51*	2	−42	28
		3.90*	2	−44	14
Insula	35	5.18*	46	16	−10
		3.71*	40	14	−16
	31	4.32*	−44	14	−8

Notes: significant primary peaks of activation in the small volume corrected areas are presented (cluster level k≥20, p<0.001); positive x-values denote right-sided activation, negative x-values denote left-sided activation;

*: peak activation is significant if family-wise error (FWE) correction is applied (p<0.05).

In general, the participants judged the body odors as being relatively weak (M = 2.85, SD = 1.31), and almost neutral in quality (M = 0.68, SD = 1.08). The level of social openness did not affect these ratings (ps>0.10). However, both intensity (r = 0.352, p = 0.046, one-sided test) and, by trend, pleasantness ratings (r = 0.300, p = 0.078, one-sided test) were related to the hemodynamic response within the caudate nucleus, with higher peak activation corresponding to judging the body odors as more intense, and more pleasant, respectively. The correlational analyses are based on n = 24 individuals after excluding n = 2 individuals scoring higher than mean plus two standard deviations on the valence ratings. Including these individuals results in correlations of r = 0.399 (p = 0.022, caudate vs. valence ratings) and r = 0.198 (p = 0.166, caudate vs. intensity ratings), respectively.

## Discussion

This study aimed to compare brain responses to human chemosensory social signals of individuals describing themselves as high in social openness (HO) with the brain responses of individuals describing themselves as low in social openness (LO). Consistent with the hypotheses, HO participants display stronger activation than LO participants in brain regions known to be involved in social perception (inferior frontal gyrus) and within the reward system (caudate nucleus). These results suggest that HO individuals perceive human body odors as subjectively important social signals associated with positive experience more readily than LO individuals. This effect, however, seems not to extend to conscious evaluation, as HO and LO individuals do not differ in their judgments of the body odors' qualitative features.

The inferior frontal gyrus has been shown to be involved in social perception, as it is activated when viewing (emotional) faces (for a review see [39], [40]), during implicit facial judgments [41] or when observing positive and negative social encounters [42]. Moreover, activity within the inferior frontal gyrus is found to be positively correlated with the level of trait empathy [43], and individuals with an empathizing rather than systemizing cognitive style show pronounced activity within the inferior frontal gyrus during a face-based mind reading task [44]. Empathy seems to be crucial for social interaction. It can be regarded as having a concept about how another individual feels, being able to take another one's perspective and, in some instances, displaying a corresponding response [45]. Hence, the concept of empathy is closely related to social openness and other social competencies. In general, the inferior frontal gyrus is discussed as a crucial part of the human mirror neuron system [46], [47]. Accordingly, in HO individuals compared to LO individuals, body odors more readily activate components of a system thought to mediate the perception and recognition of actions and emotions, which is pivotal for social cognitive functioning.

The current study is the first to report activation within the reward system in response to human chemosensory social signals (for a recent meta-analysis of basal ganglia functions see [48]). HO compared to LO individuals display a stronger hemodynamic response to body odors within the caudate nucleus. Both activation within the ventral striatum (nucleus accumbens) and the dorsal striatum (caudate nucleus, putamen) have been reported consistently in positive social interaction, indicating that reward processing and social interaction share common neural substrates [49], [50]. It has even been demonstrated that the individual disposition to social openness is positively associated with the gray matter density within the striatum [10]. Considering social perception and behavior, the caudate nucleus is discussed as a neuronal correlate of trust [51], [52] and thus to be implemented in a neuronal network that positively reinforces reciprocal altruism and cooperation [53], [54]. Moreover, it has been shown to be involved in the anticipation of positive (social) encounters in the near future [55]. Interestingly, recent research showed that socially isolated individuals show less activity within the reward system in response to people than to objects, while non-lonely individuals show the opposite response pattern [56]. The authors concluded that socially isolated individuals are less rewarded by social stimuli than non-lonely individuals, mirroring the results of the current study. Here, the social signal of human body odor seems more rewarding to individuals high in social openness, than to individuals low in social openness.

Both neural responses within the inferior frontal gyrus, and neural responses within the caudate nucleus increase in strength with rising social openness scores. These results suggest that the differences between HO and LO individuals might be driven by a linear relationship between social expertise and brain responses to social odors. However, the design underlying the current study applied a two-group approach, comparing participants either belonging to the 15.8% highest ranking or to the 15.8% lowest ranking individuals in social openness within the population. Individuals showing intermediate levels of social expertise were excluded from participation, similar to other studies comparing “experts” and “novices” (e.g. [1], [2], [4], [5]) While promising, conclusions based on the results of the correlational analyses appear somewhat limited due to “missing data” within the medium range of social expertise. The issue of a potential linear relationship between social expertise and brain responses to social odors thus needs to be addressed within upcoming research.

Across all participants, the presentation of body odors activated the fusiform cortex, the cingulate cortex, and the insular cortex. These areas are discussed as being part of specialized neuronal networks involved in the processing of chemosensory social signals, strongly overlapping with areas implicated in the processing of other socioemotional information [12], [14]. The pattern of activation observed within the current study strongly suggests that the utilized body odors were processed as social signals.

The statistical criterion for significant contrasts was set at a rather liberal level (p<0.001, uncorrected) within the current study. While this threshold is not uncommon in olfactory fMRI [57]–[59], it was basically intended to account for the exploratory nature of this study. Still, the minimum cluster size was set at a comparably conservative level of k = 20 in order to detect meaningful hemodynamic responses. Future studies with higher statistical power resulting from larger sample sizes will allow for being statistically more conservative.

The body odors were judged as relatively weak and almost neutral in quality. Several other studies have shown similar patterns of weak and even non-detectable body odors [19], [21], [60]. However, similar to the current study, these body odors reliably elicited differential central nervous processing patterns despite their weak intensity. Moreover, despite the relatively weak intensity and neutral quality, analyses revealed a positive linear relationship between peak activation within the caudate nucleus and pleasantness as well as, by trend, intensity ratings. Individuals perceiving the body odors as relatively more intense and pleasant also showed a stronger hemodynamic response within the reward system. This relationship strongly suggests that the current results indeed derive from the presentation of the body odors, and are not mainly driven by some general difference between HO and LO individuals in their response to external stimulation.

Taken together, the current results suggest that high compared to low socially open individuals tend to process human body odors as significant and valued social signals. In general, individuals describing themselves as outgoing, as having a positive attitude towards others, and as being able to show appropriate behavior in social situations, experience social interaction as considerably more rewarding than individuals describing themselves as shy, less socially competent and more socially anxious. As human body odor inherently indicates the presence of others, for individuals high in social openness it might signal the opportunity to engage in putatively appreciated social interaction. The current study, however, does not allow for concluding that this pattern is restricted to the perception of chemosensory social signals. Future research might show in how far similar effects of social expertise are observable in response to other kinds of social stimuli, such as faces or voices. Moreover, future research should examine how such differences between “social experts” and “social novices” directly affect social behavior.
